# Introductory Lecture

**Published:** 1873-11

**Authors:** 


INTRODUCTORY LECTURE. The Intr ductory Lecture to the Regular Course at the Buffalo Medical College, will be given at the College, Wednesday evening, Nov. 5th, at 7½ o'clock, by Prof. M. G. Potter. All physicians in the city and vicinity are invited to attend.

